# WATER versus WATER II 5‐year update: Comparing Aquablation therapy for benign prostatic hyperplasia in 30–80‐cm^3^ and 80–150‐cm^3^ prostates

**DOI:** 10.1002/bco2.430

**Published:** 2024-09-09

**Authors:** Mohamad Baker Berjaoui, David‐Dan Nguyen, Saud Almousa, Karim Daher, Neil Barber, Mo Bidair, Peter Gilling, Paul Anderson, Kevin C. Zorn, Gopal Badlani, Mitch Humphreys, Steven Kaplan, Ronald P. Kaufman, Dean Elterman, Mihir Desai, Claus Roehrborn, Naeem Bhojani

**Affiliations:** ^1^ Division of Urology University of Toronto Toronto Canada; ^2^ Department of Urology Centre Hospitalier de l'Universite de Montreal Montreal Canada; ^3^ Clemenceau Medical Center Beirut Lebanon; ^4^ Department of Urology Frimley Park Hospital Frimley UK; ^5^ San Diego Clinical Trials San Diego California USA; ^6^ Department of Urology Bay of Plenty District Health Board Clinical School Tauranga New Zealand; ^7^ Department of Urology Royal Melbourne Hospital Melbourne Australia; ^8^ Department of Urology Wake Forest School of Medicine Winston‐Salem North Carolina USA; ^9^ Department of Urology Mayo Clinic Phoenix Arizona USA; ^10^ Department of Urology Mount Sinai Hospital New York New York USA; ^11^ Department of Urology Albany Medical College Albany New York USA; ^12^ Department of Urology University of Southern California Los Angeles California USA; ^13^ Department of Urology UT Southwestern Medical Centre Dallas Texas USA

**Keywords:** Aquablation, benign prostatic hyperplasia (BPH), lower urinary tracts symptoms (LUTS), robotics

## Abstract

**Objective:**

This study aims to compare the long‐term outcomes of Aquablation for small‐to‐moderate (30–80 cm^3^) prostates with the outcomes for large (80–150 cm^3^) prostates at 5‐year follow up.

**Methods:**

The Waterjet Ablation Therapy for Endoscopic Resection of Prostate Tissue (WATER; NCT02505919) is a prospective, double‐blind, international clinical trial encompassing 116 patients, examining Aquablation versus transurethral resection of the prostate (TURP) for LUTS/BPH in prostates sized between 30 and 80 cm^3^. In parallel, WATER II (W‐II; NCT03123250), a prospective, multicentre, single‐arm international clinical trial, explores Aquablation outcomes in prostates ranging from 80 to 150 cm^3^. Baseline parameters and 60‐month outcomes were scrutinized using statistical analyses, including Students' *t* test, Wilcoxon tests for continuous variables, and Fisher's test for binary variables.

**Results:**

There is a significant improvement in International Prostate Symptom Score (IPSS) from baseline to 60 months in both WATER (22.9 to 7.0) and WATER II (23.2 to 6.8) (*P* = 0.852). Urinary flow rate (Qmax) increased in both groups from baseline to 60 months (WATER: 9.4 to 17.3 cc/s; WATER II: 8.7 to 17.1 cc/s) (*P* = 0.933). Immediate and sustained enhancements were observed in IPSS and Qmax. At 5 years, a notable percentage of patients in both groups were BPH medication‐free (WATER: 99%; WATER II: 94%) (*P* = 0.0517) and free from surgical retreatment (WATER: 95%; WATER II: 97%) (*P* = 0.508).

**Conclusions:**

The 5‐year follow‐up affirms that Aquablation therapy exhibits sustained outcomes, minimal irreversible complications, and low retreatment rates for treating LUTS/BPH, irrespective of prostate volume ranging from 30 to 150 cm^3^.

## 1 INTRODUCTION

Benign prostatic hyperplasia (BPH) is a prevalent condition among elderly men with increasing prevalence with age, affecting approximately 60% of men by the age of 65. BPH entails the progressive benign enlargement of the prostate gland, primarily attributable to unregulated hyperplastic growth in the epithelial and fibromuscular tissues of the transition zone and periurethral area resulting in bladder outlet obstruction (BOO).^1^

Usually, BOO manifests clinically with lower urinary tract symptoms (LUTS). First‐line therapy typically encompasses lifestyle modifications and/or medications, and the recent EAU BPH guidelines also acknowledge early surgical intervention as a viable option.^2^ While transurethral resection of prostate (TURP) is regarded as the “gold standard” for small to moderately sized prostates,^3^ other minimally invasive surgical techniques (MISTs), such as nontissue resection techniques like steam injection therapy (Rezum) and the use of implants (UroLift), are gaining increased reognition.^4^ Additionally, transurethral laser photovaporization of the prostate (PVP) is also an option.

For prostate glands exceeding 80 cm^3^, open simple prostatectomy (OSP) remains the globally acknowledged standard for surgical treatment of LUTS secondary to BPH.^5^, ^6^ However, the utilization of OSP entails an abdominal incision and hospitalization with higher risks of bleeding and a prolonged hospital stay.^7^, ^8^ Prostate enucleation has emerged as an alternative modality with superior safety profiles and a shorter hospital stay when compared with OSP.^9^ Nevertheless, prostate enucleation is characterized by a steep learning curve, operator dependency, and often necessitate a dedicated fellowship.^10^ PVP has also been utilized for the treatment of large prostate glands but requires extended surgical durations and often has a high retreatment rate (RFF).^11^ A need persists for a reliable approach with established effectiveness and safety, independent of the surgeon's expertise, to address all prostate glands regardless of their size.

Aquablation (AquaBeam System, PROCEPT BioRobotics Inc., USA) has demonstrated potential in addressing this clinical need. It was cleared by the USA Food and Drug Administration (FDA) in 2018 as a treatment for LUTS due to BPH. Aquablation combines real‐time, multidimensional imaging to enhance decision‐making and surgical planning. It leverages autonomous robotic execution following the surgeon's planning, offering an innovative approach to addressing the surgical challenges associated with larger prostate glands.

The first human study of Aquablation was published in 2016.^12^ Since that time, multiple studies have demonstrated the safety and efficacy of Aquablation, with short learning curves required to achieve effective and safe results with this technology.^13^, ^14^ Clinical trials of Aquablation have been undertaken for both small to moderately sized (30–80 cm^3^) and large (80–150 cm^3^) prostates.^15^, ^16^

The aim of this study is to build upon the existing data derived from the pooled analyses to ascertain whether the effectiveness of Aquablation remains independent of prostate size with durability though 5‐years of follow‐up. This is also to ascertain if the results are consistent with the findings demonstrated in earlier publications at 1, 2, and 3 years of follow‐up.^17^, ^18^, ^19^ To achieve this, we conducted a comparative analysis utilizing data from two distinct clinical trials: one focused on Aquablation for enlarged prostates ranging between 30 and 80 cm^3^ and the other examining the procedure for prostates sized between 80 and 150 cm^3^.

## 2 PATIENTS AND METHODS

### 2.1 Trial designs and participants

WATER (Waterjet Ablation Therapy for Endoscopic Resection of Prostate Tissue; NCT02505919) is a prospective, double‐blind, multicentre, international clinical trial comparing the safety and efficacy of Aquablation to TURP for the treatment of LUTS due to BPH. Men aged 45–80 years with moderate to severe LUTS, defined as an International Prostate Symptom Score (IPSS) ≥ 12 and maximum urinary flow rate (Qmax) ≤ 15 mL/s, were included if they had a prostate volume between 30 and 80 cm^3^, as measured by Transrectal Ultrasound (TRUS).^20^ Exclusion criteria included a body mass index (BMI) ≥ 42 kg/m^2^; history of prostate or bladder cancer, neurogenic bladder, bladder calculus or clinically significant bladder diverticulum; active infection; treatment for chronic prostatitis, diagnosis of urethral stricture, meatal stenosis or bladder neck contracture; a damaged external urinary sphincter; stress urinary incontinence; post void residual volume (PVR) > 300 mL or urinary retention; self‐catheterization use; and/or prior history of prostate surgery. Anticoagulant or bladder anticholinergic users and participants with severe cardiovascular disease were also excluded from the study. Between November 2015 and December 2016, participants from 17 centres were enrolled in the study.

WATER II (NCT03123250) is a prospective, single arm, multicentre, international clinical trial of Aquablation for the surgical treatment of LUTS/BPH in men aged 45–80 years with a prostate volume between 80 and 150 cm^3^, as measured by TRUS.^21^ Patients were enrolled at 13 sites in the United States of America and three sites in Canada between September and December 2017. Patients with catheters and those who had prior surgery were allowed to participate in WATER II. All other inclusion and exclusion criteria were the same as in WATER.

All trials were approved by local ethics committees and regulatory agencies. All subjects provided informed consent.

### 2.2 Intervention

**FIGURE 1:**
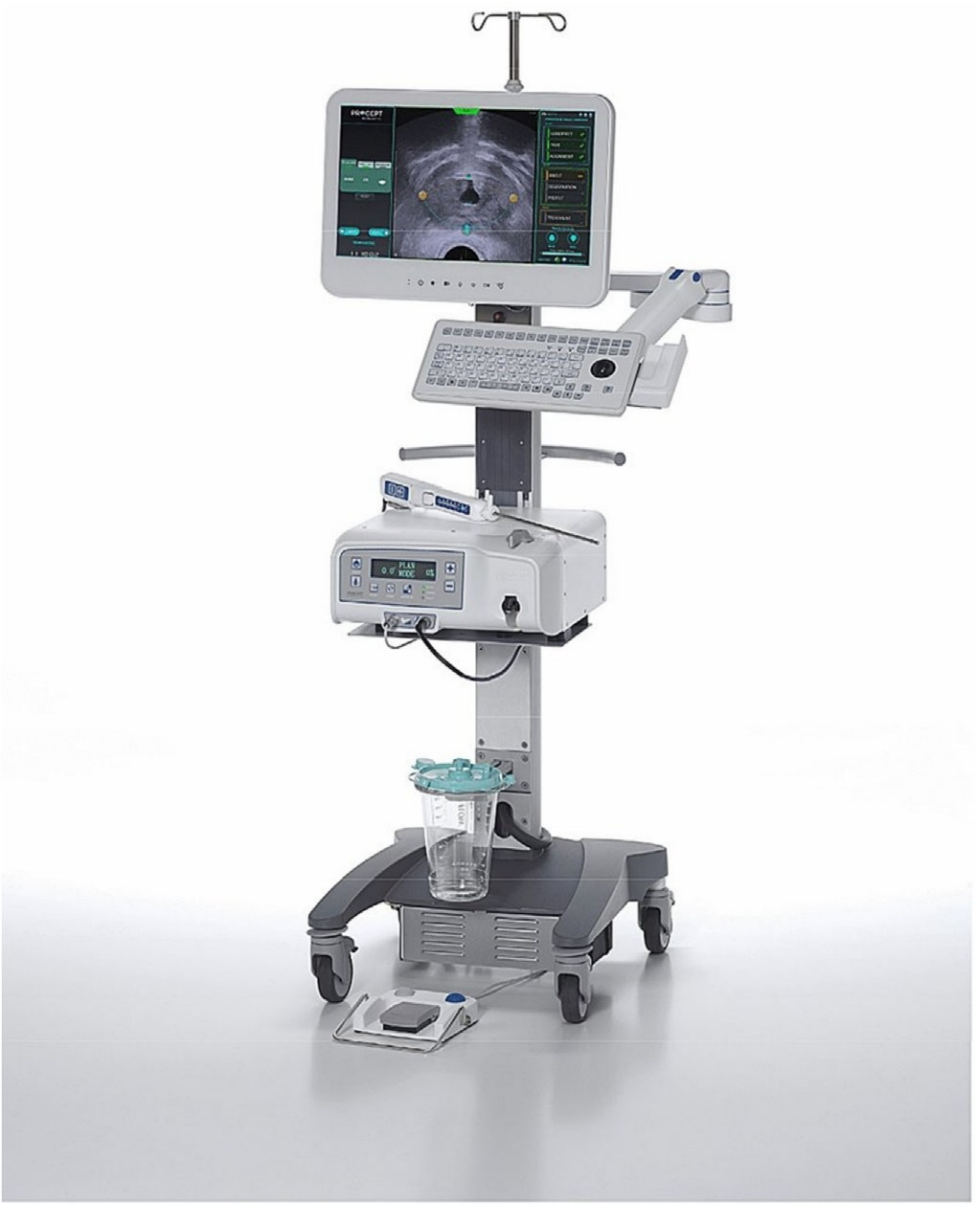
The Aquabeam system.

The Aquablation procedure was performed using the AquaBeam System as previously described (Figure 1).^12^ Following the Aquablation treatment, the bladder was thoroughly irrigated to remove residual prostate tissue and blood clots. Haemostasis was achieved using either cautery or foley tamponade. The first 46 (40%) cases performed in WATER used nonresective cautery after Aquablation. The remaining 60% utilized balloon inflation in the prostatic fossa. In WATER II, bladder neck traction using a catheter tensioning device was used without any form of cautery.

### 2.3 Study parameters

Patients completed baseline IPSS and Incontinence Severity Index (ISI) questionnaires. Uroflowmetry, PVR measurements, and standard laboratory assessment were also performed. Those questionnaires and measurements were repeated at scheduled follow‐up visits at months 1, 3, 6, 12, 24, 36, 48, and 60 months. A baseline PSA was obtained and repeated at 6 months and annually thereafter. Other questionnaires not repeated up to 60 months were not included in this analysis. Adverse events determined by the clinical events committee to be possibly, probably, or definitely related to the study procedure were assigned a Clavien–Dindo grade.

### 2.4 Statistical analysis

Student's *t* test or Wilcoxon's test was used for continuous variables and Fisher's test for ordinal/binary variables. Prostate volume was analysed as both a continuous variable and grouped consistent with study (30–80 mL for WATER vs. 80–150 mL for WATER II). Repeated measures ANOVA was used to compare responses across time points accounting for score clustering within patients. All statistical analyses were performed using the R programming language (R Foundation for Statistical Computing, Vienna, Austria). A *P* < 0.05 was considered to be statistically significant. Due to different follow‐up time periods, analyses through to month 60 are reported herein.

## 3 RESULTS

### 3.1 Baseline demographics

The WATER and WATER II trials enrolled and treated 116 and 101 patients with Aquablation, respectively. After 5 years, 58 and 60 subjects from WATER and WATER II, respectively, completed the 60‐month visit and were available for analysis. Just under half of the patients did not reach the 5‐year mark, with COVID‐19 having a significant impact due to issues including patient refusals or research department closures.^22^, ^23^ Baseline characteristics were comparable, except for prostate volume, PSA level, and a lower mean IIEF‐5 score (*P* < 0.001). The baseline demographic data is detailed in Table 1.

**TABLE 1:** Baseline characteristics.

Characteristic	WATER, Aquablation	WATER II	*P* value
	*N* = 116	*N* = 101	
Age (years)			0.0878
*N*	116	101	
Mean ± SD	65.9 ± 7.3	67.5 ± 6.6	
Median (IQR)	66.0 (60.0, 71.5)	68.0 (63.0, 72.0)	
(Min, max)	(52, 80)	(52, 79)	
Body mass index			0.8230
*P*	116	101	
Mean ± SD	28.4 ± 4.1	28.3 ± 4.1	
Median (IQR)	28.0 (25.6, 30.4)	27.5 (24.9, 30.7)	
(Min, max)	(19, 41)	(22, 41)	
Race			
Asian	2.6% (3/116)	5.0% (5/101)	0.4771
Black	1.7% (2/116)	5.9% (6/101)	0.1495
White	93.1% (108/116)	87.1% (88/101)	0.1692
Hispanic or Latino	1.7% (2/116)	.% (0/0)	
Other	0.9% (1/116)	2.0% (2/101)	0.5989
Ethnicity			
Hispanic or Latino	2.6% (3/116)	8.9% (9/101)	0.0704
Non‐Hispanic or Latino	97.4% (113/116)	91.1% (92/101)	0.0704
Prostate‐specific antigen (ng/mL)			<0.0001
*N*	116	100	
Mean ± SD	3.7 ± 3.0	7.1 ± 5.9	
Median (IQR)	2.8 (1.3, 5.0)	5.2 (2.6, 10.2)	
(Min, Max)	(0, 15)	(0, 29)	
Use of catheters 45 days prior to enrolment	.% (0/0)	15.8% (16/101)	
Prostate size TRUS (mL)			<0.0001
*N*	116	101	
Mean ± SD	54.1 ± 16.3	107.4 ± 20.2	
Median (IQR)	52.3 (40.1, 67.9)	105.0 (90.7, 120.0)	
(Min, max)	(25, 80)	(80, 150)	
Middle lobe	.% (0/0)	83.2% (84/101)	
Intravesical component	.% (0/0)	80.2% (81/101)	
Intravesical protrusion (mm)			
*N*	0	81	
Mean ± SD	. ±.	1.8 ± 0.8	
Median (IQR)	. (.,.)	1.7 (1.4, 2.1)	
(Min, max)	(.,.)	(1, 7)	
Baseline questionnaires			
IPSS Score			0.6922
*N*	116	101	
Mean ± SD	22.9 ± 6.0	23.2 ± 6.3	
Median (IQR)	24.0 (19.0, 27.5)	24.0 (18.0, 28.0)	
(Min, max)	(12, 35)	(12, 35)	
IPSS QoL			0.1809
N	116	101	
Mean ± SD	4.8 ± 1.1	4.6 ± 1.0	
Median (IQR)	5.0 (4.0, 6.0)	4.0 (4.0, 5.0)	
(Min, max)	(2, 6)	(2, 6)	
Sexually active (MSHQ‐EjD)	80.0% (92/115)	77.8% (77/99)	0.7381
MSHQ‐EjD, sexually active men			0.8993
*N*	92	75	
Mean ± SD	8.1 ± 3.7	8.1 ± 3.9	
Median (IQR)	9.0 (5.5, 11.0)	9.0 (5.0, 11.0)	
(Min, max)	(1, 15)	(1, 15)	
IIEF‐5, sexually active men			0.1984
N	91	76	
Mean ± SD	16.3 ± 7.2	14.8 ± 7.7	
Median (IQR)	19.0 (10.0, 23.0)	15.0 (7.5, 22.5)	
(Min, max)	(1, 25)	(2, 25)	
Antithrombotic use			
Anticoagulant	1.7% (2/116)	4.0% (4/101)	0.4204
Antiplatelet/NSAID including high‐dose aspirin	12.9% (15/116)	20.8% (21/101)	0.1443
Aspirin	20.7% (24/116)	17.8% (18/101)	0.6104
Any of above	35.3% (41/116)	42.6% (43/101)	0.3283
BPH medication use			
Alpha blocker	60.3% (70/116)	69.3% (70/101)	0.2009
5‐ARI	20.7% (24/116)	32.7% (33/101)	0.0630
Alpha blocker/5‐ARI	19.0% (22/116)	28.7% (29/101)	0.1090
Any of above	62.1% (72/116)	73.3% (74/101)	0.0841

### 3.2 Perioperative outcomes

Perioperative outcomes were extensively analysed in the 1‐year comparison paper.^17^ The mean procedure time was 32.8 min (standard deviation [SD] 16.5 min; range 10–96 min) in WATER and 37.4 min (SD 13.5 min; range 15–97 min) in WATER II (*P* = 0.027). The first 46 (40%) cases performed in WATER used nonresective cautery after Aquablation. The remaining 60% utilized balloon inflation in the prostatic fossa. In WATER II, bladder neck traction using a catheter tensioning device was used without any form of cautery. The mean length of stay was 1.4 days for the WATER group and 1.6 days for the WATER II group (*P* = 0.007). The mean catheter time was 2 days (SD 2.3 d; range 0.25–19 days) in WATER and 3.9 days (SD 3.6 days; range 0.7–30 days) in WATER II (*P* < 0.001).^17^

### 3.3 Functional outcomes

**FIGURE 2:**
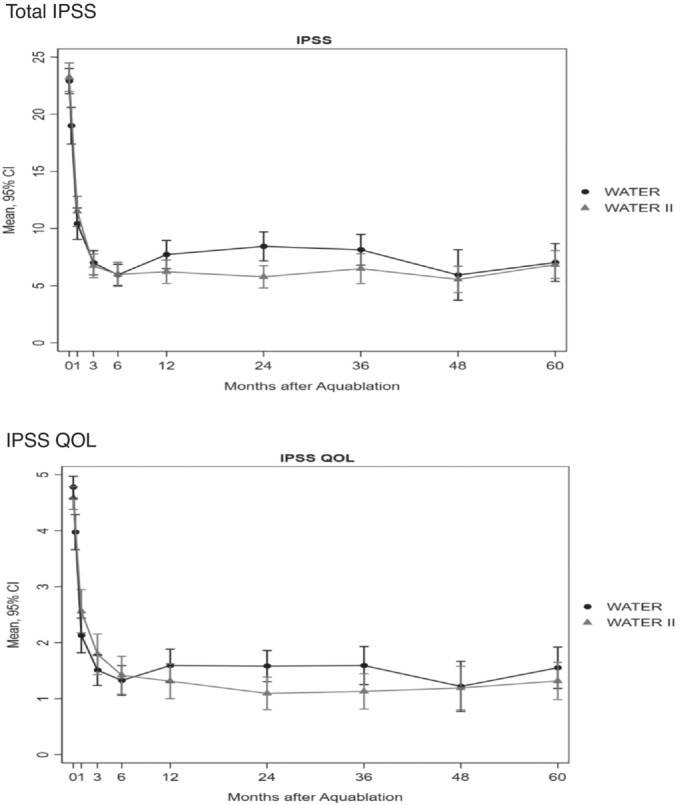
International Prostate Symptom Score (IPSS), and IPSS quality of life (QOL) scores by month after Aquablation in WATER and WATER II. CI = confidence interval.

Mean IPSS scores improved in WATER and WATER II from 22.9 and 23.2 at baseline to 7.0 and 6.8 at 60 months, respectively. The corresponding mean 60‐months improvements were 15.1 and 15.9 points; both changes were highly statistically significant (*P* < 0.0001). The mean IPSS quality of life (QOL) score improved from 4.8 and 4.6 points at baseline to 1.6 and 1.1 points at 60 months indicating an improvement of 3.2 and 3.3 points, respectively (*P* < 0.001). IPSS scores are presented in Figure 2.

**FIGURE 3:**
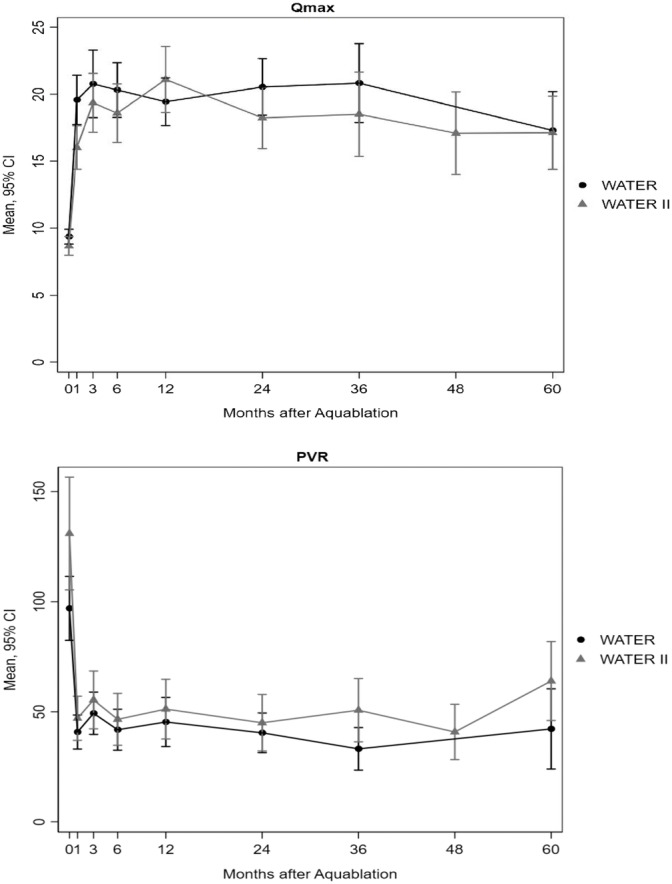
Qmax and PVR cores by month after Aquablation in WATER and WATER II.

Uroflowmetery measures also demonstrated improvement. Mean Qmax improved from 9.4 and 8.7 cm^3^/s at baseline in WATER and WATER II to 17.3 and 17.1 cm^3^/s at 60 months, representing improvements of 8.7 and 9.2 cm^3^/s, respectively (*P* < 0.001). Mean PVR decreased from 97 and 131 cm^3^ to 42 and 64 cm^3^ at 60 months (decrease of 55 and 67 cm^3^; *P* < 0.001), respectively. Uroflowmetry results are presented in Figure 3.

### 3.4 Retreatment rates, PSA, and PV changes

**FIGURE 4:**
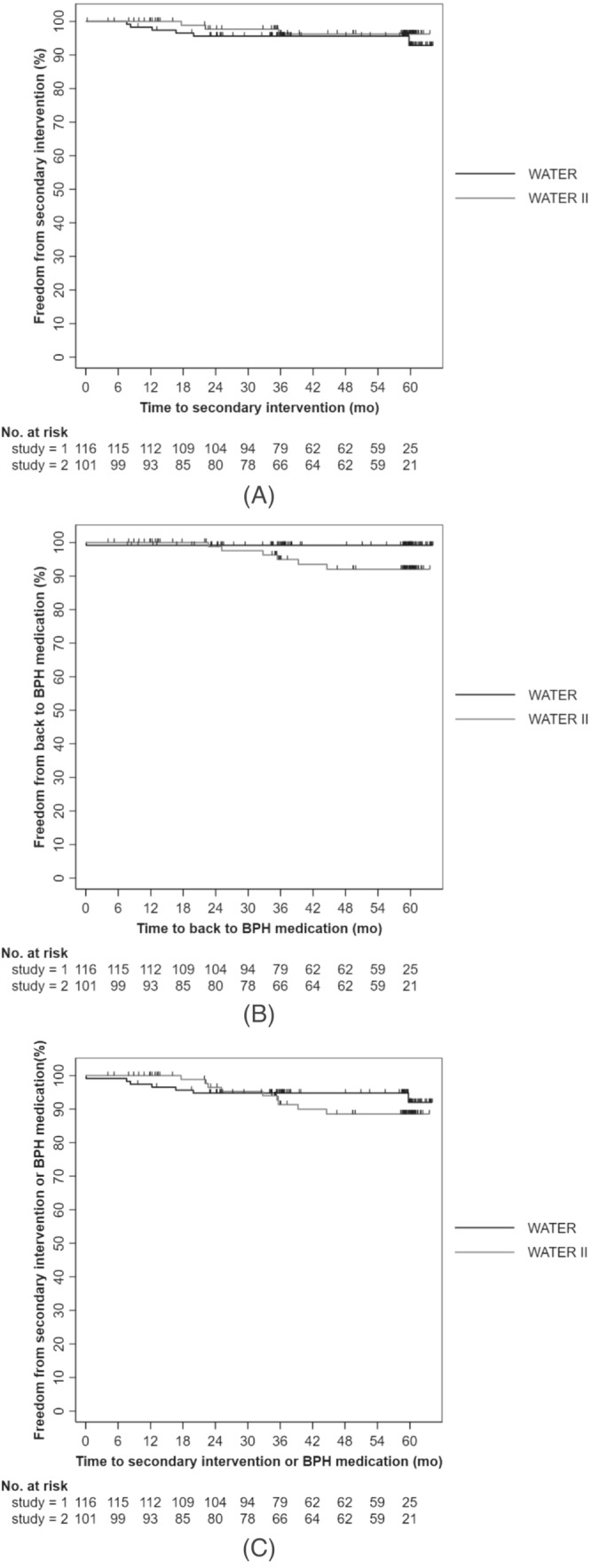
(A) Freedom from secondary intervention due to LUTS over 5 years. (B) Freedom from requiring to be back on BPH medical therapy due to LUTS over 5 years after Aquablation. (C) Freedom from requiring secondary surgical intervention or being back on BPH medical therapy due to LUTS over 5 years after Aquablation.

**FIGURE 5:**
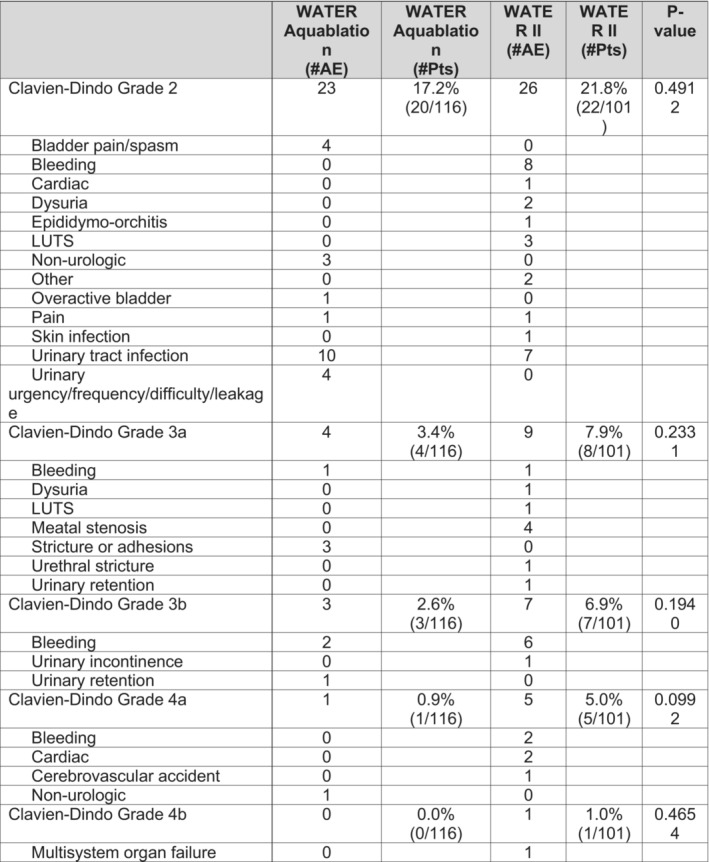
Distribution of events at month 6 categorized by Clavien–Dindo grades (possibly, probably, or definitely related).

At 5 years, the Kaplan–Meier freedom from surgical retreatment was 92.8% in WATER and 96.2% in WATER II, with only six and three patients, respectively, requiring surgical retreatment for BPH. When the populations were combined the Kaplan–Meier freedom from surgical retreatment was 94.3%. Kaplan–Meier freedom from medical BPH retreatment (defined as initiation of an α blocker or 5‐α reductase inhibitor after Aquablation) at 5 years 99.1% (*n* = 1) of patients in WATER and 92.0% (*n* = 6) in WATER II. When the populations were combined the Kaplan–Meier freedom from medical BPH retreatment was 95.7%. Figure 4A–C shows the Kaplan–Meier surgical retreatment‐free survival curve, BPH medications‐free curve, and combined surgical and BPH medications‐free curves, respectively. Surgical retreatment rate was defined as requiring surgery for BPH and thus any treatment for urethral strictures was not accounted for, but was clearly specified in Clavien Dindo complications that happened upon to 6 months after treatment (Figure 5).

**FIGURE 6:**
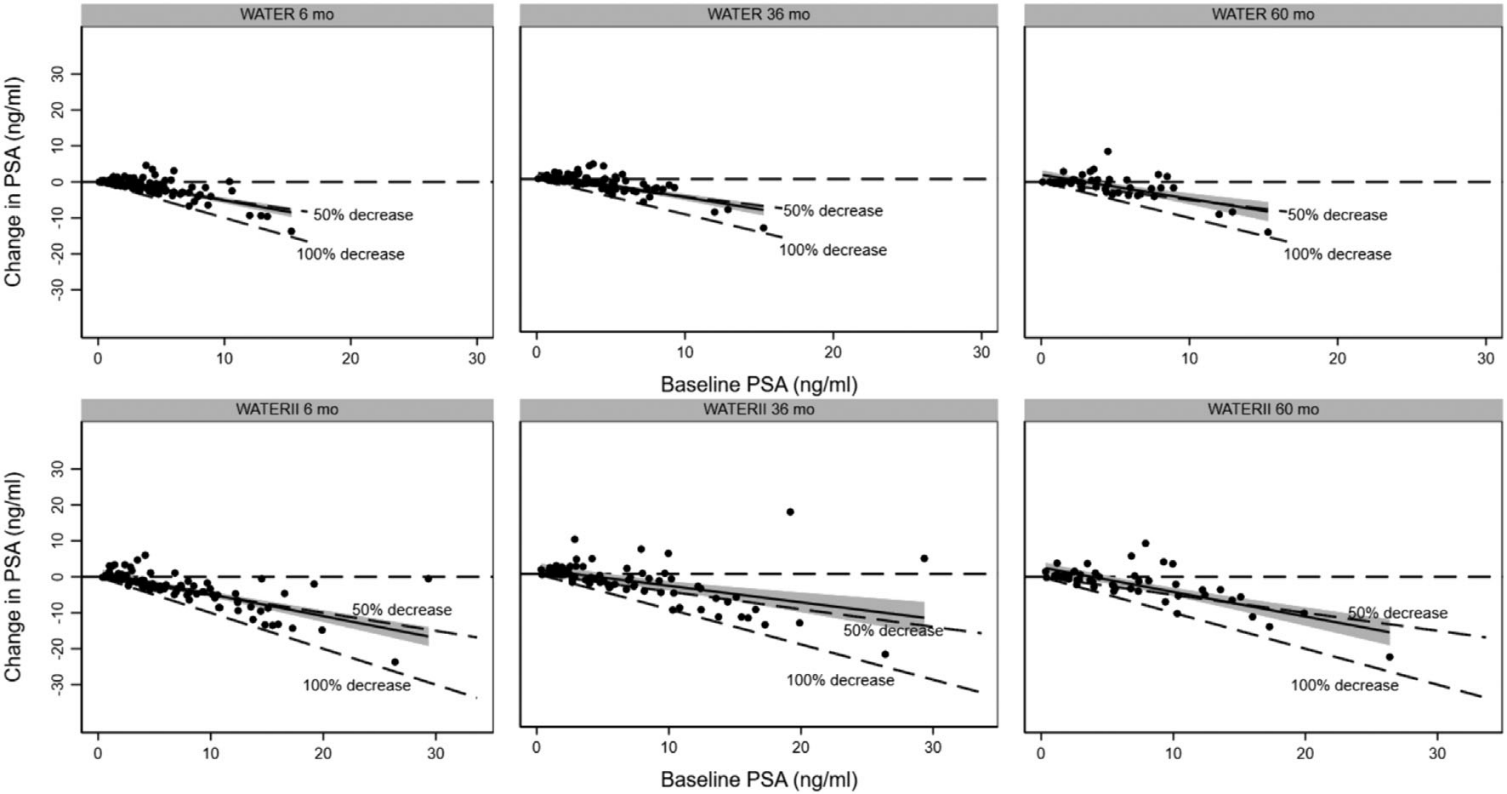
Change in PSA at 6 months, 3 years, and 5 years.

**FIGURE 7:**
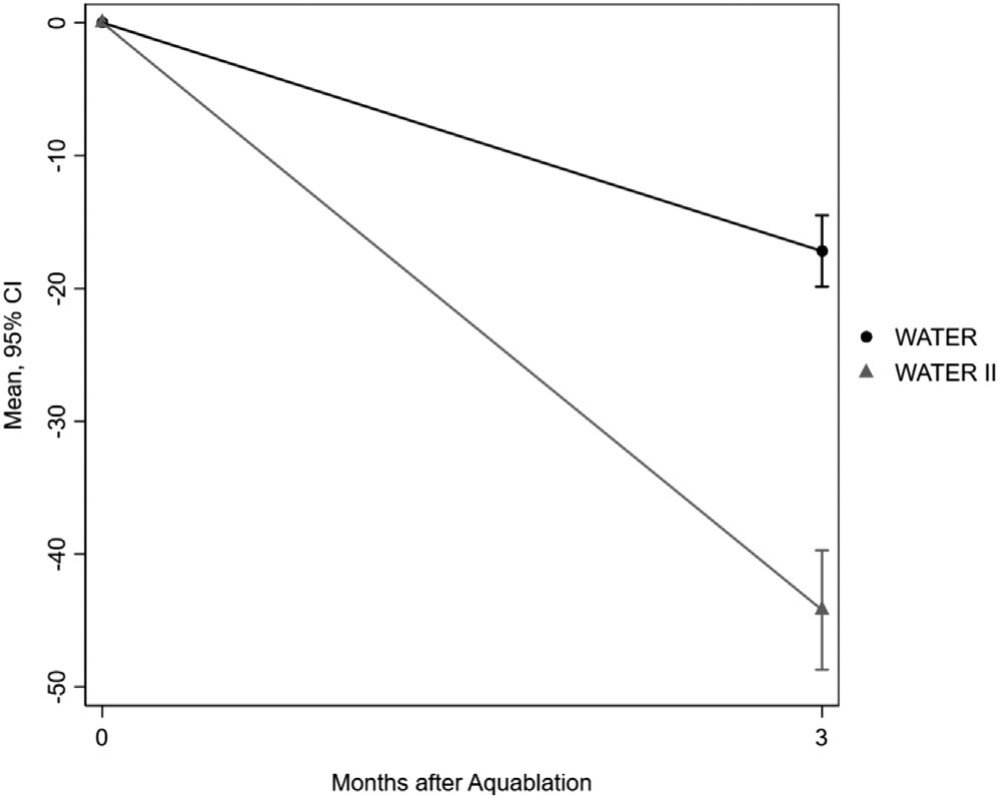
Change in prostate size.

Regarding changes in PSA, baseline PSA was 3.7 ng/mL in WATER and 7.1 ng/mL in WATER II; at 5 years, PSA was 3.5 and 4.7 ng/mL, respectively. Figure 6 represents the change in PSA at 6, 36, and 60 months; the regression line is at or below the 50% reduction line for all time points. Furthermore, the prostate volumes (PV) were recorded for patients in both Water and Water II at the beginning of the trial and at 3 months. PV in WATER decreased from 54.1 to 37.2 cc and in WATER II from 107.4 to 63.1 cc at 3 months. Figure 7 shows change in PV.

## 4 DISCUSSION

Our current analysis affirms the enduring clinical advantages of Aquablation for LUTS secondary to BPH in small‐to‐moderate sized prostates (30–80 cm^3^) extending its applicability to large‐to‐very‐large prostates (80–150 cm^3^) up to 5 years. This is of particular significance for prostates exceeding 80 cm^3^, where there is a heightened demand for a surgical approach that combines efficacy and durability with prostate volume independence as well as reproducibility of outcomes irrespective of the surgeon, and a less invasive alternative to OSP.

The outcomes in these studies did not necessitate highly experienced surgeons in Aquablation. Remarkably, among the 17 sites in WATER, 14 had no prior experience. Similarly in WATER II, of the 16 sites, nine had no prior experience with Aquablation at the trial's commencement.^17^

No statistically significant differences in baseline characteristics were observed between the two cohorts, except for factors related to prostate volume, such as PSA. Notably, there was a 5‐min increase in the time from the insertion of the ultrasound probe to the catheter insertion, along with an additional 4 min of resection time for larger prostates.^17^ This extended operative time aligns with expectations and remains considerably shorter compared with alternative surgical modalities, attributed to the efficiency and precision inherent in the robotically executed procedure.^24^

Similar trends in IPSS, IPSS QOL, and Qmax have been observed across both trials. Notably, in both cohorts, the results appear to exhibit durability through 5‐years, but no further improvement was noted over the last 12 months. This aligns with the anticipated natural history of BPH, which tends to recur over time, possibly attributed to prostate enlargement over 5 years. However, it is noteworthy that the retreatment rates, whether surgical or medical, remain consistently below 10% in both cohorts. These rates, even after 5 years, are comparable or even lower than those reported for TURP,^23^ Urolift,^25^ Rezum,^26^ and GreenLight PVP.^27^, ^28^ In contrast, retreatment rates are slightly higher than the retreatment rates associated with prostate enculeation.^9^, ^29^

Laser enucleation continues to be regarded as the only available option to treat BPH regardless of the prostate volume.^30^ However, its utilization is limited by the steep learning curve and a significant dependence on surgeon proficiency. Typically, endoscopic enucleation methods necessitate 20–40 cases to learn the procedure and more to master it.^31^ Conversely, a prospective multicentre evaluation of the learning curve found nearly half of the centres abandoned HoLEP due to operating difficulty and complications.^32^

This underscores the significance of Aquablation as a BPH treatment method, independent of prostate volume, and particularly valuable due to its minimal surgeon‐to‐surgeon reproducibility. Aquablation reduces outcome variability by relying on the surgeon's surgical planning, as the procedure is guided, automated, and executed robotically, offering live ultrasound imaging throughout.

Our study had some limitations; for instance, the WATER II is a single‐arm study without a control group, although a performance goal was used, preventing direct comparisons with other techniques. Additionally, while we present evidence of the durability of our previous findings at the 5‐year follow‐up, more extended‐term data from these trials are imperative to validate the volume‐independent durability of the treatment outcomes.

The impact of COVID‐19 on the patient follow‐up warrants addressing. We compared the patient follow‐up in WATER and WATER, which were 50% and 59%, respectively, to two other contemporary FDA clinical studies that have reached 5‐year follow‐up, the LIFT study and the Rezum pivotal trial. Their percentage of patients reaching the 5‐year visit were 62%^25^ and 57%,^26^ respectively. While the follow‐up rates of WATER II are similar to other FDA clinical trials, the authors believe the additional cohort analysis to show no difference in baseline and IPSS outcomes instils confidence in the overall study results and conclusions.

Furthermore, the decrease in PSA approached 50% during the 5 year interval and reached 33% at 5 years as compared with HoLEP, which maintained a 65% drop in PSA at 5 years.^33^ This difference is expected when comparing enucleation to Aquablation.

Future studies are warranted to compare Aquablation to other modalities for the surgical treatment of large prostates. A prospective randomized clinical trial, WATER III (NCT04801381), is currently underway in Europe to compare Aquablation with enucleation in large prostates. Quintas et al compared enrolment of 100 subjects, comparing Aquablation and enucleation at 6 months.^34^ Both groups exhibited similar baseline characteristics, including an average prostate size of approximately 78 mL. No transfusions occurred in either arm, and at the 6‐month mark, IPSS, IPSS QoL, Qmax, and PVR were comparable between the two therapies. Notably, ejaculatory dysfunction was 98% for enucleation and 0% for Aquablation. Despite the concern of dropout rates from WATER and WATER II, real time data is showing a repetition of the results from those two trials. For example, a recent study by Omidele et al. looking at 4 years of real‐world Aquablation data showed results that were very comparable to the WATER and WATER II trials.^35^

## 5 CONCLUSIONS

Aquablation therapy consistently achieves normalized clinical outcomes across diverse prostate sizes and shapes. The notable benefits of Aquablation include reduced operative times, short learning curves, and favourable clinical outcomes regardless of prostate size. Most notably, these benefits are sustained over a 5‐year follow‐up period.

## AUTHOR CONTRIBUTIONS

*Manuscript generation*: Mohamad Baker Berjaoui, David‐Dan Nguyen, Saad Almousa, and Karim Daher. *Data collection and quality review*: Neil Barber, Mo Bidair, Peter Gilling, Paul Anderson, Kevin C. Zorn, Gopal Badlani, Mitch Humphreys, Steven Kaplan, Ronald P. Kaufman, Jr., Dean Elterman, Mihir Desai, Claus Roehrborn, and Naeem Bhojani.

## CONFLICT OF INTEREST STATEMENT

Mo Bidair worked as a consultant for improving current machine at Procept BioRobotics. Dean Elterman worked as an investigator and consultant at Procept BioRobotics. Kevin Zorn worked as consultant at Procept BioRobotics, consultant at Boston Scientific, and consultant at Laborie. Mitchell Humphreys worked as a consultant at Applaud, BD Peripheral, Boston Scientific, and Vitruoso. Mitchell Humphreys received a grant from Boston Scientific. Mihir Desai worked as a consultant for Procept BioRobotics. Kevin Zorn and Naeem Bhojani were paid for a training session at AUA 2018. Peter Gilling, Neil Barber, and Paul Anderson have performed commercial Aquablation procedures. No other author has conflict of interest with PROCEPT BioRobotics.
